# Collection of free-living accelerometry data in large clinical studies before and during the COVID-19 pandemic

**DOI:** 10.1093/geroni/igab046.3571

**Published:** 2021-12-17

**Authors:** Jacek Urbanek, Kunihiro Matsushita, Robert Kaplan, Josef Coresh, Jennifer Schrack

**Affiliations:** 1 Johns Hopkins School of Medicine, Baltimore, Maryland, United States; 2 Johns Hopkins University, Baltimore, Maryland, United States; 3 Albert Einstein College of Medicine, New York, New York, United States

## Abstract

The recent COVID-19 pandemic had a substantial impact on clinical research, including recruitment and follow-up visits in new and ongoing studies, especially affecting ones focused on older, at-risk adults. As the objective assessment of physical activity with wearables is usually initiated during in-person visits, the collection of these data experienced substantial, unplanned gaps. We report the frequency of data collection (visits-per-month) in studies collaborating with the Accelerometry Resource Core (ARC) at Johns Hopkins Center on Aging and Health. We focus on two, NIH-funded, studies that implemented the ARC accelerometry protocol. The Atherosclerosis Risk in Communities that stopped visit 8 enrollment in early 2020 and reinstated in 2021 for visit 9, and the Peripheral Artery Disease Study of SOL that started the data collection in early 2021, first via the mail-in protocol, then shifting towards in-clinic visits. Through March 2020, ARC processed an average of 125 new accelerometry per month (SD = 54). There was no new data collected for the remainder of 2020. The collection restarted in January 2021 with an average of 55 (SD = 43) files a month in the first and 112 (SD = 53) in the second quarter of 2021. A total of 573 new accelerometry observations were collected across both studies since the first wave of COVID-19 in March 2020 including 282 observations collected exclusively using a mail-in protocol. This recovery of data collection demonstrates that wearable devices allow for safer, remote assessment of physical activity, function, and sleep eliminating the need for in-person visits.

